# Functional Consequences of Mutations and Polymorphisms in the Coding Region of the PAF Acetylhydrolase (PAF-AH) Gene

**DOI:** 10.3390/ph2030094

**Published:** 2009-11-20

**Authors:** Diana M. Stafforini

**Affiliations:** Huntsman Cancer Institute and Department of Internal Medicine, University of Utah, Salt Lake City, UT 84112, USA; Email: diana.stafforini@hci.utah.edu; Tel.: +1-801-585-3402; Fax: +1-801-585-0101.

**Keywords:** PAF acetylhydrolase, PLA_2_G7, single nucleotide polymorphism, mutation, vascular disease

## Abstract

In the past several years a number of alterations in the PAF-AH/PLA_2_G7/LpPLA_2_ gene have been described. These include inactivating mutations, polymorphisms in the coding region, and other genetic changes located in promoter and intronic regions of the gene. The consequences associated with these genetic variations have been evaluated from different perspectives, including * in vitro* biochemical and molecular studies and clinical analyses in human subjects. This review highlights the current state of the field and suggests new approaches that can be used to evaluate functional consequences associated with mutations and polymorphisms in the PAF-AH gene.

## 1. Introduction

The first step in the metabolism of two classes of inflammatory phospholipid mediators, Platelet-Activating Factor (PAF) and oxidatively-fragmented phospholipids (OxPL), is catalyzed by enzymatic activities known as PAF acetylhydrolases (PAF-AHs). This family of phospholipases A_2_ includes intracellular and secreted activities. The secreted form of PAF-AH (also known as lipoprotein-associated phospholipase A_2_, LpPLA_2_, PLA_2_G7) circulates in plasma as a complex with high- and low-density lipoproteins (HDL and LDL, respectively). While the physiological consequences of dual lipoprotein association remain to be completely identified, it is apparent that the location of the enzyme impacts its function and that aberrant distribution is associated with a variety of syndromes. The role of PAF-AH in human biology continues to be the subject of intense research as increasing numbers of laboratories around the world are utilizing complementary approaches to examine the function of this enzyme *in vivo*. The well-recognized fact that altered expression of PAF-AH correlates with the incidence and severity of vascular and other inflammatory diseases has sparked the interest of a wide range of scientists and clinicians in both the public and private sectors. In addition, recent elegant work by Vadas and co-workers showed direct correlations between serum PAF levels and severity of anaphylaxis [1]. Importantly, PAF-AH activity was inversely correlated with disease severity and it was significantly lower in patients with fatal anaphylactic reactions than in patients in the control groups [1]. Considerable effort has been devoted to evaluating whether genetic variations in the PAF-AH gene are associated with the incidence and severity of human diseases, and whether these alterations affect the levels of protein expression and/or its function. A number of PAF-AH loss-of-function mutations and single nucleotide polymorphisms (SNPs) have been identified and studied to various extents with the objective of elucidating their impact in physiology and disease. These analyses have provided new mechanistic and clinical insights that may have important translational utility, including the potential for personalized diagnostic and therapeutic applications. 

## 2. Naturally Occurring Genetic Alterations in the PAF-AH Gene

**Figure 1:**
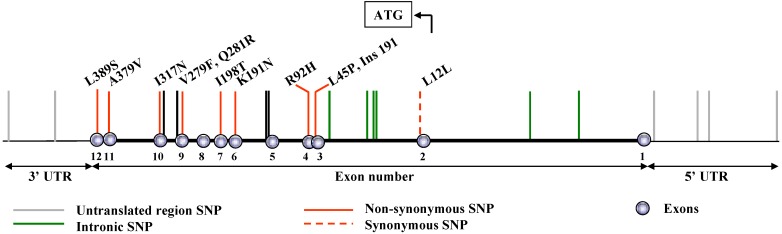
Diagrammatic representation of mutations and polymorphisms in the PAF-AH gene.

In humans, the PAF-AH gene harbors a number of mutations and polymorphisms within coding and non-coding regions (Figure 1). 

The consequences of these genetic variations, particularly those that alter protein sequence, have been investigated in considerable detail in terms of the frequency with which they occur in both healthy and diseased subjects. One of the most thoroughly investigated variants is a loss-of-function mutation that occurs near the active site, and that has been observed mainly in Japanese [2], Taiwanese [3], Korean [4], and Chinese [5] populations. Three additional, but much less prevalent, loss-of-function mutations have been reported in Japanese subjects [6,7]. In addition, the coding region of the PAF-AH gene harbors several SNPs that moderately impact enzymatic activity of the gene product; these will be discussed in subsequent sections. Genetic alterations have also been described in untranslated regions [8]. This article summarizes current information on the incidence and functional consequences of non-synonymous variants in the coding region of the gene. A discussion of SNPs located in promoter, intronic, and 3’-untranslated regions of PAF-AH is beyond the scope of this review.

## 3. Inactivating Mutations

PAF-AH deficiency was initially characterized by Miwa and co-workers using functional approaches [2]. This trait is quite prevalent in Japan, as approximately 4% of the population lacks expression of the activity in plasma [2]. In most cases, PAF-AH deficiency occurs as a consequence of a missense mutation near the active site that results in a valine to phenylalanine transition (V279F, [9]). A much less frequent mutation, Q281R, also results in severe deficiency of PAF-AH [6,10]. Studies by Ishihara and co-workers revealed the occurrence of two additional loss-of-function variants, the incidence of which seems quite rare [7]. While PAF-AH deficiency alone has not been reported to have major physiological effects, several laboratories have demonstrated that a phenotype becomes evident when partial or complete deficiency is combined with other genetic defects or environmental factors, as is the case for many other gene products. Genetic studies in healthy and diseased populations indicate that PAF-AH deficiency affects the incidence and severity of a number of inflammatory diseases. 

### 3.1. V279F (rs 16874954)

Most subjects who express virtually no plasma PAF-AH activity harbor two F279 alleles; heterozygous subjects express half the normal plasma activity [9]. Molecular studies revealed that the recombinant mutant protein is inactive [9]. The fact that a bulky residue (phenylalanine) replaces a smaller amino acid (valine) near the active site of the enzyme likely accounts for the loss of function associated with this mutation [9]. While the initial discovery of V279F was made in a Japanese cohort, the mutation was subsequently reported in Korean [4], Taiwanese [3], and Chinese [5] populations, and in subjects from Turkey, Azerbaijan, and Kyrgyzstan [11]. Allele frequency varies widely in these populations: the highest prevalence was reported in Japan and in Taiwan, followed by Korea and China (Table 1). Our studies, and those of others [12,13,14], revealed that complete PAF-AH deficiency does not occur in Caucasian subjects, and this has been confirmed in public databases. This variant likely originated in Japanese and Korean ancestors. The clinical consequences associated with complete and partial PAF-AH deficiency have been evaluated in patients suffering from a variety of inflammatory conditions in the vascular, respiratory, renal, and digestive systems (Table 1). These analyses clearly demonstrated that the incidence of the F279 allele correlates with several manifestations of vascular disease in Japanese subjects; multiple studies by Yamada and co-workers and by Unno *et al.* formed the bases to support this conclusion (Table 1).

**Table 1:** The V279F (rs16874954) polymorphism: allele frequency in control and diseased populations.

Population	Clinical Status	n	Allele frequency, %	p	Authors [Ref.]
Japanese	Controls	263	17.9		Stafforini *et al.* [10]
	Severe asthma	266	22.6	**0.02**	
	Control adults	188	21.0		
Japanese	Control children	142	20.0		Ito *et al.* [15]
	Asthmatic children	118	31.0	**0.007-0.004**	
Japanese	Controls	217	21.7		N. Satoh *et al.* [16]
	Asthma	279	18.6	N/S	
Japanese	Controls	106	12.7		Unno *et al.* [17]
	Abdominal aortic aneurysm	131	21.4	**0.012**	
Japanese	Controls	106	12.7		Unno *et al.* [18]
	Femoropopliteal bypass	50	21.4	**<0.001**	
Japanese	Controls	158	19.0		Unno *et al.* [19]
	Peripheral artery occl. disease	150	13.0	**0.031**	
Japanese	Controls	114	13.0		Unno *et al.* [20]
	Atherosclerotic occl. disease	104	20.0	**<0.05**	
	Controls	222	12.6		
Japanese	Non fam. dilated cardiomyop.	122	21.3	**0.003**	Ichihara *et al.* [21]
	Controls	1,684	17.0		
Japanese	Risk for atherosclerosis	1,398	18.0	**<0.001**	Yamada *et al.* [22]
	Atherosclerosis	850	22.0	**0.0019**	
Japanese men	Controls	452	12.7		
Japanese women	Myocardial infarction	373	18.6	**0.0002**	Yamada *et al.* [23]
	Controls	150	16.3		
	Myocardial infarction	81	25.9	N/S	
	Controls	284	16.0		
Japanese	Nonfamilial hypertrophic cardiomyopathy	142	27.0	**0.004**	Yamada *et al.* [24]
Korean	Controls	670	14.0		Jang *et al.* [4]
	CVD	532	10.2	**0.005**	
	Controls	909	5.6		
Chinese Han	CHD	808	5.0	N/S	Hou *et al.* [5]
	Myocardial infarction	502	5.2		
Turkish	Controls	128	1.3		Sekuri *et al.* [25]
	Premature CAD	115	0	N/S	
Taiwanese	Controls	200	17.0		Liu *et al.* [3]
	Myocardial infarction	200	16.0	N/S	
Japanese	Controls	134	14.2		Hiramoto *et al.* [26]
	Stroke	120	23.8	**0.01**	K. Satoh [27]
	Controls	270	15.6		
Japanese	Cerebral hemorrhage Hypertension	99	24.2	**<0.01**	Yoshida *et al.* [28]
		138	19.9	N/S	
Chinese Han	Controls	215	11.2	**Statistically**	Zhang *et al.* [29]
	Cerebral Infarction	102	19.0	**significant**	
Japanese	Type 2 diabetes, 40-59 y/o	50	14.0	**Statistically**	Yamamoto *et al.* [30]
	Type 2 diabetes, 60-79 y/o	50	30.0	**significant**	
Japanese	Controls	100	16.0		Tanaka *et al.* [31]
	IgA nephropathy	89	17.0	N/S	
	Controls	100	16.0		
Japanese	Steroid responsive nephrotic syndrome	101	12.0	N/S	Xu *et al.* [32]
Japanese	Controls	100	16.0		Xu *et al.* [33]
	Hemolytic uremic syndrome	50	15.0	N/S	
	Controls	11	0		
Caucasian	Uncomplicated infection with *E. coli* O157:H7	52	0	N/A	Smith *et al.* [14]
	Hemolytic uremic syndrome	15	0		
Japanese	Control	108	17.0		Nakamura *et al.* [34]
	Ulcerative colitis	53	22.5	N/S	
Japanese	Control	188	21.0		Ohtsuki *et al.* [35]
	Schizophrenia	191	19.0	N/S	
	Controls	213	14.8		
Japanese	Conventional MS	151	12.6	N/S	Osoegawa *et al.* [36]
	Opticospinal MS	65	16.9	N/S	
Japanese	Control	106	15.6		Minami *et al.* [37]
	Kawasaki disease	76	13.2	N/S	
Caucasian	Randomly selected	1,202	0	N/A	Schnabel *et al.* [13]

This Table summarizes results from studies in various human populations genotyped for the V279F polymorphism. The incidence of rs16874954 is reported in terms of the percentage with which the allele was represented in healthy and diseased subjects. The “n” column refers to the number of participants in each study category and includes subjects harboring 0-1-2 polymorphic alleles. The “p” column depicts whether statistically significant differences were found in polymorphism incidence in control and diseased populations, where available/appropriate. CAD: coronary artery disease; CHD: coronary heart disease; CVD: cardiovascular disease; MS: multiple sclerosis; N/A: not applicable; N/S: Not statistically significant.

A study by Yamamoto and co-workers demonstrated that the intima media thickness in Japanese type 2 diabetics who harbored the F279 allele was significantly greater than that observed in age-matched patients who expressed wild-type PAF-AH [30]. This study suggested that F279 was associated with the development of carotid atherosclerosis in elderly diabetics. Wang and co-workers reported a significant increase in the oxLDL/LDL ratio in homozygous deficient subjects [38], suggesting that complete PAF-AH deficiency increases the susceptibility of LDL to oxidation, as previously shown in purified *in vitro* systems [39]. 

Some of these observations, however, were not confirmed in other laboratories. Reports by Liu *et al*. and by Hou and co-workers in Taiwanese and Chinese patients reported no association of F279 with myocardial infarction or CHD [3,5]. The relatively low frequency of the mutant allele in the Chinese population may explain results by Hou *et al.*, as correctly pointed out by the authors [5]. In addition, the fact that neither study evaluated the results based on gender may have prevented the authors from identifying potential correlations. This is an important issue when one considers that PAF-AH deficiency has been shown to be more common in Japanese male, but not female, patients diagnosed with coronary artery disease, compared to healthy controls [23]. A study in Korean subjects reported that the frequency of F279 in this population is similar to that observed in Japan, and that this allele is less frequent in Korean men diagnosed with cardiovascular disease (Table 1, [4]). The reasons for this discrepancy are unknown, but it is possible that the criteria used for patient selection differed from those utilized in other studies. 

Studies in Kei Satoh’s laboratory demonstrated that the incidence of F279 is associated with stroke and cerebral hemorrhage in Japanese subjects [26,27,28]. Interestingly, Zhang and co-workers also reported that F279 may be an independent risk for atherosclerotic cerebral infarction in Chinese subjects [29], suggesting that the clinical impact of F279 is not limited to Japanese populations. Studies in the respiratory system revealed similar trends. We found that the incidence and severity of asthma was higher in Japanese PAF-AH deficient subjects compared to healthy controls [10]. This finding was confirmed by another group [15] but not by N. Satoh *et al*. [16]. A limitation of the study by N. Satoh *et al.* is that the prevalence of the F279 allele in control subjects was higher than that detected in most other reports in Japanese populations (Table 1). It is possible that the higher-than-normal frequency of F279 in the controls accounted for the observations reported in this study. 

Additional work in patients afflicted by a variety of diseases revealed potential contributions of F279 to the severity, but not the incidence, of a number of syndromes. In these analyses, F279 was shown to contribute to disease progression rather than onset. First, while F279 was reportedly unrelated to either susceptibility or severity of conventional multiple sclerosis, it increased the severity of female opticospinal multiple sclerosis [36]. Second, Nakamura and co-workers found no significant differences in genotypic frequency of ulcerative colitis patients compared to controls [34]. However, unresponsiveness to steroid therapy was significantly higher in patients who harbored one F279 allele compared to those who did not (66.7 vs. 27.6 percent, p = 0.019). The authors concluded that F279 could be a useful marker to predict responsiveness to steroid therapy [34]. Third, no significant differences have been detected in genotype frequency between Kawasaki Disease patients and controls [37]. However, patients who harbored one or two F279 alleles required additional intravenous immunoglobulin administration (52% vs 14%, p = 0.001). Three studies reported that F279 correlates with the severity of renal diseases (Table 1). Tanaka and co-workers showed that IgA nephritic children who carried the F279 allele excreted more protein in the urine compared to patients who harbored only the wild-type allele [31]. In addition, the percentage of glomeruli with mesangial cell proliferation was significantly greater in F279 carriers, suggesting that the mutation may increase the severity of childhood IgA nephropathy [31]. Second, patients diagnosed with steroid-responsive nephritic syndrome and who harbored one F279 allele had more relapses during the first year after disease onset compared to patients who carried only the wild-type allele. This observation suggests that genotyping at this locus could facilitate identification of children likely to have a disease relapse [32]. Finally, the mean duration of oligoanuria was significantly longer in heterozygous compared to homozygous wild- type patients diagnosed with *E. coli* O157-associated hemolytic uremic syndrome (p = 0.012, [33]). Most (75%) heterozygous subjects required dialysis, while most (63%) patients who harbored only the wild-type allele did not. Interestingly, aberrant PAF production has been reported in hemolytic uremic syndrome patients. A study in Caucasians who did not harbor F279, showed a rise in PAF levels following infection with *E. coli* O157, and decreased levels of the mediator during development of the disease [14]. It is possible that the initial increase in PAF levels is followed by resolution without full disease development in subjects who can rapidly and effectively inactivate PAF. Unfortunately, this study did not assess the levels of PAF-AH activity, so it is not possible to draw firm conclusions at this point. It is tempting to speculate, however, that subjects deficient in PAF-AH activity may have more severe forms of hemolytic uremic syndrome owing to impaired abilities to inactivate PAF. In summary, results from independent studies indicate that the presence of F279 increases the severity of several renal disorders and that identification of subjects who harbor this mutation could have significant clinical value.

### 3.2. Q281R, I317N and Ins 191

A relatively uncommon mutation (Q281R) located two amino acids downstream of the most prevalent inactivating PAF-AH mutation was shown to result in severe, but not complete, loss of enzymatic activity ([6,10], Figure 1). The precise incidence of this variant is likely quite low, even in Japanese populations. Previous studies suggested an allele frequency of approximately 1.0 % for this variant [7,40]. The Q281R mutation is near the active site of PAF-AH and is therefore likely to affect proper folding in the region, thus impairing enzymatic activity. A woman who harbored two R281 alleles suffered from coronary artery disease and hypertension [6]. In addition, a patient harboring F279 and R281 alleles had severe asthma [10]. Finally, Kujiraoka *et al.* identified a diabetic subject who was heterozygous for the Q281R mutation [40]. These isolated observations are consistent with findings in subjects harboring the F279 allele and they suggest that impaired PAF-AH function correlates with the incidence of several human diseases.

Ishihara and co-workers described two rare mutations in the coding region of PAF-AH. An insertion of adenine at nucleotide 191 in exon 3 (Ins191) creates a premature termination at codon 63 and impairs PAF-AH function ([7], Figure 1). In addition, a novel mutation in which adenine is substituted for thymine at nucleotide 950 in exon 10, results in substitution of N for I at codon 317 (I317N, Figure 1). This mutation could theoretically create a new N-linked glycosylation site on PAF-AH, but this has not been confirmed experimentally. Ins191 and I317N impair PAF-AH secretion by macrophages isolated from subjects carrying the mutant alleles [7]. 

### 3.3. Consequences associated with complete versus partial deficiency of PAF-AH

An issue that merits consideration is related to the functional consequences associated with partial versus complete deficiency of PAF-AH. In certain settings, partial deficiency of the enzymatic activity may result in a disease phenotype intermediate between that of subjects harboring two wild-type alleles and that of individuals harboring two mutant alleles. However, this may not always be the case. In fact, a 50% reduction in PAF-AH levels such as that typical of subjects harboring one F279 allele, may not significantly affect the extent of substrate hydrolysis [41]. When substrate levels are relatively low (*i.e.*, nanomolar range), the rate-limiting step of the reaction is likely determined by factors other than total enzyme activity levels. Instead, the type of lipoprotein with which the enzyme associates, substrate levels, accessibility of substrates to the active site, and/or their solubility may determine the extent of hydrolysis [41]. A corollary of this observation is that *partial* deficiency of PAF-AH due to genetic, pharmacologic, or other causes may have limited functional consequences *in vivo*. Wang and co-workers correctly pointed out that it may not be appropriate to jointly analyze heterozygous and homozygous deficient subjects in clinical studies aimed at assessing the consequences of PAF-AH deficiency [38]. To rigorously determine the impact of aberrant PAF-AH expression in human diseases, it will be essential to independently compare cohorts of subjects harboring 0, 1, and 2 functional PAF-AH alleles.

## 4. Other Non-Synonymous and Synonymous Amino Acid Substitutions

In contrast to V279F, the incidence of which seems limited to defined populations, three relatively well studied non-synonymous polymorphisms [R92H, I198T, and A379V (Figure 1, Table 2)] occur in all populations studied so far. Affinities of the purified variants for PAF are summarized in Table 2.

**Table 2:** Affinity of wild-type, human PAF-AH and several variants for PAF.

Construct	Amino acid at position 92	Amino acid at position 198	Amino acid at position 379	Km μM [Kruse et al., (42)]	Km, μM (our work)
Wild type	R	I	A	7.0	14.5
R92H	H	I	A	9.0	12.0
I198T	R	T	A	42.0	24.0
A379V	R	I	V	14.0	14.0
R92H-I198T	H	T	A	11.0	28.9
R92H-A379V	H	I	V	8.0	12.0
I198T-A379V	R	T	V	50.0	30.0

### 4.1. R92H (rs1805017)

**Figure 2:**
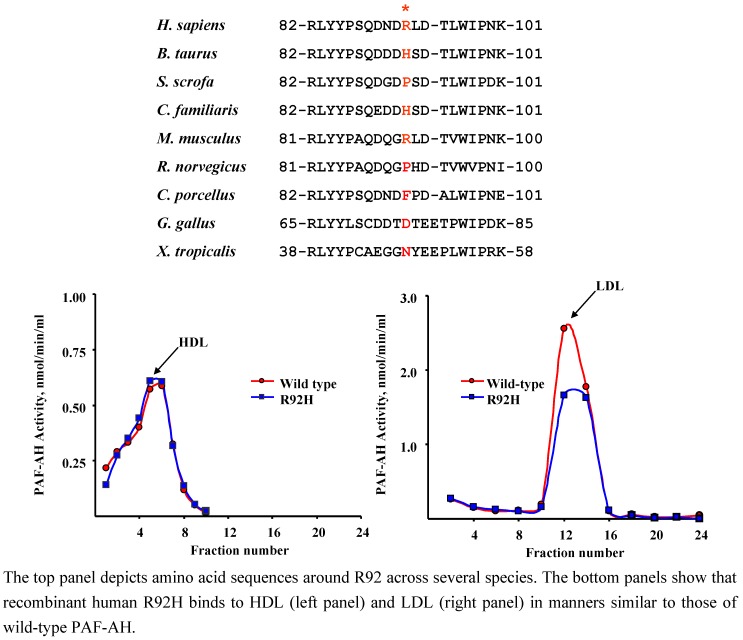
The R92H variant binds to human HDL and LDL *in vitro.*

The presence of arginine at codon 92 as the major allele in plasma PAF-AH is observed only in humans and in mice (Figure 2). This indicates that absolute conservation of this residue is not essential for proper function. The role played by the amino acid present at this position has been analyzed in a variety of subjects from different populations. The largest study conducted thus far (3234 subjects) detected modest but statistically significant decreases in plasma activity levels in German subjects harboring 1 or 2 H92 alleles [43]. Similar results were recently reported by Li *et al.* who analyzed a cohort of ARDS patients [44]. In contrast, studies in Chinese and European subjects revealed no effects of H92 on total plasma PAF-AH activity [5,45]. These observations are consistent with kinetic studies by Kruse *et al*. [42] who expressed, purified, and tested the recombinant mutant protein. These investigators found that purified preparations of R92H and wild-type PAF-AH displayed similar affinities for PAF and hydrolyzed the substrate at comparable rates. While susceptibility of the mutant to substrate inhibition was much higher than that of the wild-type protein, it is clear that this was not a reflection of increased substrate affinity. Our own studies revealed that the presence of histidine at position 92 does not affect binding of PAF-AH to LDL or HDL *in vitro* (Figure 2, [46]), and does not alter substrate affinity (Table 2). 

These combined observations indicate that the presence of histidine at position 92 of PAF-AH has modest or no impact on optimized plasma activity levels, does not significantly affect classic biochemical properties of the protein, and does not seem to alter location. However, it would be of importance to complement these studies with lipoprotein distribution profiles in fresh plasma samples from subjects that harbor 0-1-2 H92 alleles as these analyses would definitively establish whether H92 contributes to the ability of PAF-AH to bind to lipoprotein particles *in vivo.*

Genetic studies revealed that the allele frequency of H92 varies among populations. A method of base-quenched probe for polymerase chain reaction genotyping that requires only a pair of primers and one fluorescent probe was recently described for the detection of this polymorphism [47]. In public databases, the lowest frequency (13.5%) has been reported in a Japanese cohort and the highest prevalence (35.0%) was observed in a primarily Caucasian cohort (http://www.ncbi.nlm.nih.gov/). Clinical studies centered on the contribution of H92 to disease incidence revealed similar frequencies of this polymorphism in healthy subjects compared to atopic and asthmatic patients (Table 3, [42]) and in patients with schizophrenia (Table 3, [12]). In addition, there seem to be no changes in the frequency of this polymorphism in patients with coronary artery diseases or myocardial infarction, compared to healthy subjects.

**Table 3:** The R92H (rs1805017) polymorphism: allele frequency in control and diseased populations.

Population	Clinical Status	n	Allele frequency	p	Authors [Ref.]
British	Controls	150	23.2		Kruse *et al.* [42]
	Atopic asthma	150	23.4	N/S	
German	Nonatopic	33	24.1	N/S	
	Atopic	72	25.4	(IgE levels)	
British	Controls	146	24.7		Bell *et al.* [12]
	Schizophrenia	298	25.2	N/S	
Caucasian	Controls	693	25.8		Hoffmann *et al.* [43]
	CAD	2,541	26.0	N/S	
German	Controls	484	22.4		Ninio *et al.* [45]
	CAD	1,303	27.1	**0.015**	
Caucasian + African American + American Indian	Controls	267	34.0		Sutton *et al.* [8]
	CAD (< 56y/o)	599	28.0	**0.01-0.04**	
	CAD (> 56 y/o)	207	21.0	**0.0001-0.0002**	
	Myocardial infarction	425	28.0	**0.0008-0.002**	
Chinese	Controls	896	17.2		Hou *et al.* [5]
	CHD	806	18.2	N/S	
	Myocardial infarction	499	20.1	N/S	
Caucasian	ARDS	41	20.7	N/A	Li *et al.* [44]
African American	ARDS	17	29.4	N/A	
Caucasian	Controls	355	27.5		Limou *et al.* [48]
	AIDS (slow progressors)	168	26.8	N/S	
	AIDS (rapid progressors)	54	29.6	N/S	
Japanese	Randomly selected	1,878	21.1	N/A	Kokubo *et al.* [49]
Caucasian	Randomly selected	1,183	26.8	N/A	Schnabel *et al.* [50]

This Table summarizes results from studies in various human populations genotyped for the R92H polymorphism. The incidence of rs1805017 is reported in terms of the percentage with which the allele was represented in healthy and diseased subjects. The “n” column refers to the number of participants in each study category and includes subjects harboring 0-1-2 polymorphic alleles. The “p” column depicts whether statistically significant differences were found in polymorphism incidence in control and diseased populations, where available/appropriate. AIDS: acquired immune deficiency syndrome; ARDS: acute respiratory distress syndrome; CAD: coronary artery disease; CHD: coronary heart disease; N/A: not applicable; N/S: not statistically significant.

Two exceptions include a study by Ninio *et al.* describing increased risk of coronary artery disease associated with H92 [45] and a report by Sutton *et al*. [8] that found the opposite trend, that is, a protective effect of the minor allele (Table 3). The suitability of control cohorts utilized in these two studies has been questioned owing to the fact that the frequency of the H92 allele (22.4 and 34%, respectively) differed from that observed in other studies in similar populations (Table 3) and in an unaffected cohort (n=718) of GENECARD [43]. In addition, the report by Sutton *et al.* indicated that no differential gene expression was observed in diseased arteries and showed no influence of the polymorphism on disease burden in the aorta [8]. Given that a larger recent study [43] failed to confirm the contradictory associations reported in these analyses [8,45], it is tempting to conclude that H92 does not modulate, or has a modest impact, on vascular disease. 

Recent work by Li and co-workers [44] showed that ARDS patients carrying the H92 allele were mechanically ventilated for a longer time period when compared to non-carriers. This patient group expressed 25.8% lower plasma PAF-AH activity compared to patients harboring the wild-type allele. This interesting study also reported increased survival in patients who expressed higher plasma PAF-AH levels seven days following diagnosis. The results indicate that inter-individual variability in plasma PAF-AH activity may have prognostic value and suggest that PAF-AH genotyping and assessment of plasma activity levels in critically ill patients may help in the selection of therapeutic approaches that can be tailored to the needs of individual patients. 

### 4.2. I198T (rs 1805018)

**Figure 3:**
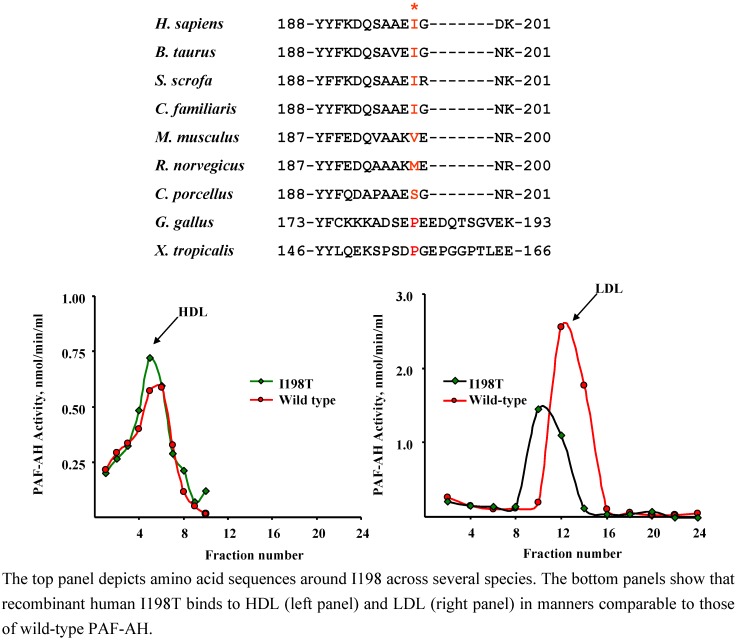
The I198T variant binds to human HDL and LDL *in vitro.*

Individuals harboring a threonine at position 198 of PAF-AH were first described by Bell and co-workers [12]. The degree of conservation of this residue across species is moderate (Figure 3). Expression of the recombinant variant revealed that enzymatic activity was preserved [42]. However, the mutant protein displayed decreased affinity for PAF (Table 2, [42]). Our studies revealed that the presence of T198 did not affect binding to LDL or HDL *in vitro* (Figure 3, [46]). As in the case of R92H, it would be of interest to complement these studies with lipoprotein distribution profiles in fresh plasma samples from subjects that harbor 0-1-2 T198 alleles, as these analyses would establish whether T198 contributes to the ability of PAF-AH to associate with lipoprotein particles *in vivo.*

Individuals homozygous for the I198T polymorphism are rare, so most of the work in humans has been conducted in heterozygous subjects. There is controversy on the impact of T198 on the levels of expression of plasma PAF-AH protein and activity. Three investigations revealed no statistically significant effect of T198 on enzymatic activity [43,44,45]. However, a recent study by Hou *et al*. showed that subjects harboring this variant expressed substantially lower levels of PAF-AH activity [5]. The haplotypes of these subjects differed from those of the control subjects in more than the I198T polymorphism [5] and it is not clear whether these additional genetic variations contributed to the observed decreases in activity. This is particularly important as PAF-AH risk alleles may have an additive effect that leads to the generation of dysfunctional protein products [8]. Sutton *et al.* reported a trend towards decreased PAF-AH expression in the aorta of subjects harboring T198 [8]. These observations leave open the possibility that at least in some populations, T198 affects activity and/or protein levels, or protein stability, in addition to its effect on substrate affinity.

Public databases (http://www.ncbi.nlm.nih.gov/) report that the lowest T198 allele frequency occurs in Caucasian (4.2–12.0%) and Asian (4.3–11.0%, excluding Japanese) cohorts. The frequency is much higher in Japanese subjects (19.7%), in African Americans (23.5%), and in sub-Saharan African populations (28.5%). 

Kruse and co-workers found the T198 variant to be highly associated with total IgE concentrations in an atopic population and with atopic asthma in an asthmatic population (Table 4, [42]). Interestingly, T198 seems to be somewhat more prevalent in ARDS patients [44] compared to control populations with similar ethnic backgrounds (Table 4), suggesting that additional, well-controlled studies related to the impact of T198 on ARDS incidence and severity may be warranted.

The prevalence of the T198 polymorphism has been found to be weakly associated with the incidence of schizophrenia ([12], Table 4). Mechanistically, it is possible that the variant contributes to these syndromes as a consequence of decreased protein expression, at least in some populations [5]. Alternatively, since substrate concentration and/or accessibility to the active site tend to be rate limiting *in vivo* [41], decreased substrate affinity (*i.e,* increased Km [42]) may account for the observed effects. 

A variety of studies have assessed the incidence of T198 in patients with cardiovascular disease (Table 4). These analyses have led to conflicting results possibly owing to the size and heterogeneity of the patient populations and/or the type of statistical analyses utilized. Sutton *et al.* used logistic regression modeling and found no significant association of T198 with CAD outcome [8]. Similarly, Ninio *et al.* reported that the incidence of cardiovascular events was not significantly associated with survival outcome by univariate analyses [45]. In contrast, Hoffmann and co-workers reported lower incidence of T198 in a CAD cohort. The larger size of the populations studied by Hoffmann *et al.* may explain why this correlation did not become evident in other studies. Kokubo and co-workers reported that the presence of T198 was associated with hypertension in women when the data were adjusted for several factors and analyzed by multivariate logistic regression analyses [49]. In summary, while there is no absolute agreement on the precise role of the somewhat rare T198 polymorphism on activity and/or disease incidence, it seems that additional large-scale studies should further evaluate whether, and how, T198 contributes to inflammatory and vascular disorders. These analyses should include activity measurements as at least one study [5] showed a remarkably high inhibitory effect of this polymorphism on PAF-AH levels. It seems that analyses in Japanese cohorts would be ideal for this purpose, owing to the reportedly high prevalence of the polymorphism in this population.

**Table 4:** The I198T (rs1805018) polymorphism: Allele frequency in control and diseased populations.

Population	Clinical Status	n	Allele frequency,%	p	Authors [Ref.]
British	Controls	150	7.6		Kruse *et al.* [42]
	Atopic asthma	150	11.7	**0.008**	
German	Nonatopic	33	0.8		
	Atopic	72	3.7	**0.0087** (IgE levels)	
British	Controls	99	2.5		Bell *et al.* [12]
	Schizophrenia	204	6.4	**0.04**	
Caucasian	Controls	693	6.1		Hoffmann *et al.* [43]
	CAD	2,541	4.4	**0.009**	
German	Controls	484	5.7		Ninio *et al.* [45]
	CAD	1,311	5.4	N/S	
Caucasian + African American + American Indian	Controls	267	6.0		Sutton *et al.* [8]
	CAD (< 56y/o)	599	9.0	N/S	
	CAD (> 56 y/o)	207	7.0	N/S	
	Myocardial infarction	425	8.0	N/S	
Chinese	Controls	909	8.7		Hou *et al.* [5]
	CHD	808	9.8	N/S	
	Myocardial infarction	502	9.9	N/S	
Caucasian	ARDS	41	9.8	N/A	Li *et al.* [44]
African American	ARDS	17	11.8	N/A	
Caucasian	Randomly selected	1,202	5.2	N/A	Schnabel *et al.* [13]
Japanese	Randomly selected	1,878	20.7	N/A	Kokubo *et al.* [49]
Japanese	Controls	96	28.1	N/A	Jinnai *et al.* [51]
Caucasian	Randomly selected	1,202	5.2	N/A	Schnabel *et al.* [13]

This Table summarizes results from studies in various human populations genotyped for the I198T polymorphism. The incidence of rs1805018 is reported in terms of the percentage with which the allele was represented in healthy and diseased subjects. The “n” column refers to the number of participants in each study category and includes subjects harboring 0-1-2 polymorphic alleles. The “p” column depicts whether statistically significant differences were found in polymorphism incidence in control and diseased populations, where available/appropriate. ARDS: acute respiratory distress syndrome; CAD: coronary artery disease; CHD: coronary heart disease; N/A: not applicable; N/S: not statistically significant.

### 4.3. A379V (rs 1051931)

**Figure 4:**
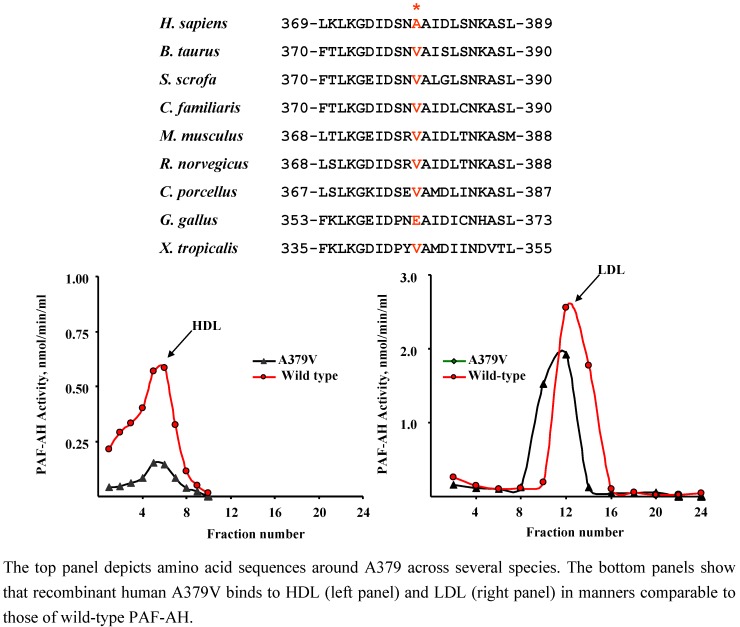
The A379V variant binds to human HDL and LDL *in vitro.*

Individuals harboring a valine at position 379 of PAF-AH were described a few years ago [12]. While humans express predominantly an alanine residue at this position, most species harbor a valine residue (Figure 4). This feature forecasts limited impact of V379 on enzyme function in humans. Purified recombinant A379V displays increased maximal velocity and a two-fold decrease in substrate affinity compared to the wild-type protein ([42], Table 2). We found that the A379V variant associated with LDL and HDL in fashions comparable to those of the human protein (Figure 4, [46]). However, a systematic study to characterize the lipoprotein distribution of PAF-AH in subjects homozygous for the V379 polymorphism has not, to my knowledge, been performed.

As in the case of other polymorphisms, there is controversy on the impact of V379 on plasma PAF-AH protein and activity levels. Hoffmann and co-workers reported increases in PAF-AH plasma levels with a clear gene-dose effect [43]. Similarly, Ninio and collaborators found that the presence of V379 was significantly associated with increased PAF-AH activity in a co-dominant fashion (p = 0.02, [45]). Schnabel and co-workers reported higher PAF-AH activity in subjects carrying two V379 alleles and extended the observation to a large community-based cohort [50]. While the observed differences were statistically significant in these two instances, the increases in activity were modest (<10 %). Three unrelated studies found no statistically significant effect of V379 on enzymatic activity in ARDS patients (Table 5), in a Chinese cohort, and in a Caucasian control group [5,44,52]. An interesting study in a Taiwanese population showed dramatic decreases in activity in subjects homozygous for the V379 allele [3]. The fact that this is the only study reporting such large effects suggests that an additional factor(s) peculiar to this group of subjects likely contributed to the results. In summary, the presence of V379 may confer a very modest increase in plasma PAF-AH activity levels in some populations. These effects are likely mediated by the enzyme itself, because studies that evaluated properties of the purified recombinant A379V variant in a lipoprotein-free assay system recapitulated the increased enzymatic activity that was observed in some clinical studies [42]. 

**Table 5:** The A379V (rs1051931) polymorphism: allele frequency in control and diseased populations.

Population	Clinical Status	n	Allele frequency	p	Authors [Ref.]
British	Controls	150	15.2	**0.038**	Kruse *et al.* [42]
	Atopic asthma	150	21.6		
German	Nonatopic	33	10.3	**0.0017**	
	Atopic	72	21.9		
German	Controls	484	24.3	**0.0007**	Ninio *et al.* [45]
	CAD	1298	19.5		
Caucasian + African American + American Indian	Controls	267	15.0		Sutton *et al.* [8]
	CAD (< 56y/o)	599	20.0	0.05	
	CAD (> 56 y/o)	207	26.0	**0.002**	
	Myocardial infarction	425	19.0	0.01	
Taiwanese	Controls	200	21.0	**0.01**	Liu *et al.* [3]
	Myocardial infarction	200	33.0		
Caucasian	Controls	693	20.9	N/S	Hoffmann *et al.* [43]
	CAD	2541	21.4		
Chinese	Controls	904	15.9		Hou *et al.* [5]
	CHD	808	16.6	N/S	
	Myocardial infarction	503	15.5	N/S	
Korean	Controls	670	14.6	N/S	Jang *et al.* [4]
	CVD	532	15.5		
European	Controls	556	24.0	--	Abuzeid *et al.* [53]
	Myocardial infarction	527	22.0		
Caucasian	Male controls	359	20.2		Wootton *et al.* [52]
	Male CHD	104	21.2	N/S	
	Female controls	244	20.9		
	Female CHD	50	23.0	N/S	
Caucasian	Early ARDS	41	13.4	N/A	Li *et al.* [44]
African American	Early ARDS	17	11.8	N/A	
British	Controls	93	25.8		Bell *et al.* [12]
	Schizophrenia	191	18.8	0.06	
British	Controls	123	21.5	N/A	Wootton *et al.* [54]
British Caucasian	Controls	2695	19.6	N/A	Rudd *et. al.* [55]
Mixed	Randomly selected	8105	19.4	N/A	Schnabel *et al.* [50]
Dutch	Randomly selected	3575	19.0	N/A	Van den Berg *et al.* [56]
Japanese	Randomly selected	1,878	10.8	N/A	Kokubo *et al.* [49]
Japanese	Controls	96	4.2	N/A	Jinnai *et al.* [51]

This Table summarizes results from studies in various human populations genotyped for the A379V polymorphism. The incidence of rs1051931 is reported in terms of the percentage with which the allele was represented in healthy and diseased subjects. The “n” column refers to the number of participants in each study category and includes subjects harboring 0-1-2 polymorphic alleles. The “p” column depicts whether statistically significant differences were found in polymorphism incidence in control and diseased populations, where available/appropriate. ARDS: acute respiratory distress syndrome; CAD: coronary artery disease; CHD: coronary heart disease; CVD: cardiovascular disease; N/A: not applicable; N/S: not statistically significant.

The prevalence of the V379 allele varies among populations. The lowest frequency (9.2–9.5%) has been reported in Japanese populations and the highest prevalence (26.7–32.6%) was observed in Sub-Saharan African subjects. A number of groups have investigated whether V379 is associated with the incidence of various diseases and/or physiological responses (Table 4). Bell and co-workers found no significant differences in allele frequency between patients with schizophrenia and control subjects, although a trend (p = 0.057) towards increased incidence in the patients was noted [12]. In contrast, V379 was found to be highly associated with specific sensitization in a German atopic population and with atopic asthma in a British cohort (Table 5, [42]). Wootton and co-workers reported that after ten weeks of intensive physical exercise, individuals homozygous for the V379 allele showed decreased percentage of adipose tissue compared to AV and AA genotype groups (p = 0.01, [52]). A potentially interesting observation by Li and co-workers is that ARDS patients seem to have a lower incidence of the V379 polymorphism compared to similar Caucasian cohorts [13.4% versus 19.6–25.8% in control Caucasian subjects, excluding reference [42], Table 5]. Unfortunately, this analysis only evaluated ARDS patients, so a comparison with healthy control subjects can only be made across studies. Nonetheless, the fact that ARDS survivors expressed higher plasma PAF-AH activity [44], combined with the fact that the prevalence of the V379 allele was lower in at least one ARDS cohort, suggests that V379 may offer some protection from this disease. 

Eight studies investigated whether V379 contributes to vascular disease [3,4,5,8,43,45,52,53]. Four of these analyses reported no statistically significant differences in allele frequency of control subjects compared to patients with coronary heart disease or myocardial infarction (Table 5). Three studies found statistically significant, but opposite, relationships between these parameters [3,8,45]. The suitability of control cohorts utilized in two of these reports [8,45] was questioned by others [43], as indicated above. The lack of consensus among these analyses could be due to the criteria utilized for patient selection, combined with the size and nature of the patient populations studied. The mechanism by which potential V379-mediated effects contribute to inflammatory diseases could include direct effects of the polymorphism on enzymatic activity, but this contribution is likely to be relatively modest [45]. The possibility that V379 is in linkage disequilibrium with an un-identified etiological variant cannot be ruled out.

### 4.4. L12L (rs 35142331), L45P (rs 45521937), K191N (rs 45454695), and L389S (rs 34159425)

These variants have only been reported in public databases and they occur with various frequencies in human populations. One of the variants (rs 35142331) results in no amino acid change and is thus unlikely to impact protein function. The frequency of the remaining variants is either unknown or very rare in Caucasians. The functional consequences associated with these polymorphisms are currently unknown. 

## 5. Additional Measurements May Be Necessary to Elucidate Functional Consequences of PAF-AH Polymorphisms

### 5.1. Rate of hydrolysis at sub-saturating substrate levels and effect of lipoprotein environment

**Figure 5:**
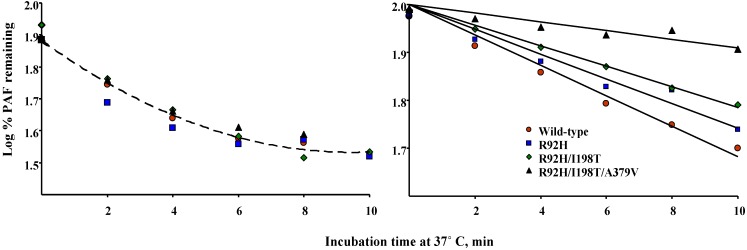
Kinetics of PAF (10^-9^ M) hydrolysis by free (left panel) and LDL-associated (right panel) PAF-AH variants.

We previously showed that the kinetics of PAF hydrolysis are dependent on the ratio between enzyme and substrate, and that at low substrate levels the rate of hydrolysis deviates from that predicted by Michaelis-Menten kinetics [41]. We investigated the effect of sequentially introducing naturally occurring PAF-AH polymorphisms on the kinetics of PAF hydrolysis at sub-saturating substrate levels. This analysis revealed that single, double, and triple mutants hydrolyzed PAF with similar efficiencies compared to the wild-type protein (Figure 5, left panel). 

In these assays, we utilized equal amounts of PAF-AH activity as determined by our standard, optimized, enzyme assay. Thus, the results were qualitatively comparable in both assay types, that is, we observed no major differences in the rate of PAF hydrolysis by PAF-AH variants. However, the experimental design in these studies did not recapitulate the environment in which the enzyme functions *in vivo*. This is an important issue because lipoproteins alter the efficiency of hydrolysis of phospholipid substrates, presumably by modulating access to the active site, altering substrate presentation, or changing solubility. To investigate this, we incorporated wild-type and PAF-AH variants into LDL, and then assessed the ability of the lipoprotein-associated variants to hydrolyze sub-saturating levels of PAF. Our studies showed that sequential introduction of each polymorphism increased the half–life of PAF in LDL (Figure 5, right panel). These results suggested that in experimental settings modeled to mimic conditions likely to occur *in vivo*, that is, when substrate is limited, enzyme levels are in excess, and the enzyme functions in its natural environment, factors other than absolute enzyme levels impact the rate of hydrolysis. These studies revealed potentially important functional consequences associated with naturally occurring polymorphisms in PAF-AH. In addition, the results illustrate the importance of complementing traditional biochemical analyses with assays that take into consideration properties peculiar to the gene product and reaction being evaluated.

### 5.2. Susceptibility to oxidative inactivation

**Figure 6:**
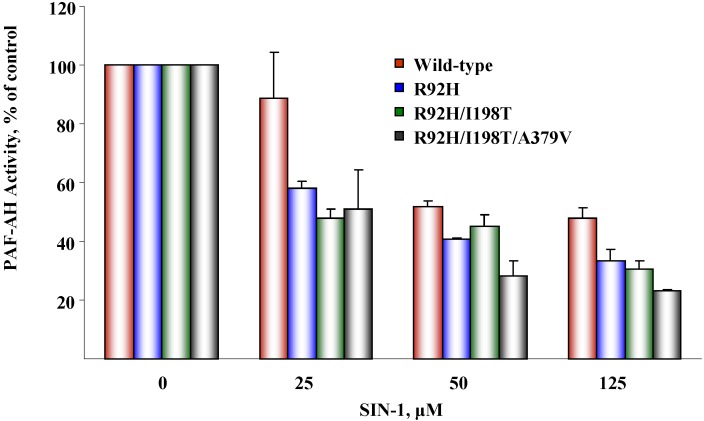
Susceptibility of PAF-AH variants to oxidative inactivation mediated by SIN-1

Previous work demonstrated that PAF-AH is susceptible to inactivation by oxidants, and that key methionine and tyrosine residues confer susceptibility to oxidant attack [57]. We investigated whether naturally occurring PAF-AH variants were differentially affected by oxidants, as this could provide additional mechanistic insight into the functional consequences of genetic alterations. We expressed single, double, and triple mutants harboring R92H, I198T, and A379V, and then investigated if these polymorphic forms of PAF-AH were differentially affected by SIN-1, a peroxynitrite-generating agent [57]. We found that sequential replacement of these residues increased susceptibility to oxidative inactivation (Figure 6). These findings suggested that subjects harboring these polymorphisms may accumulate PAF-AH substrates in settings of elevated oxidant stress that are characteristic of acute inflammation and other syndromes. 

## 6. Conclusions

In this article, I have presented current information that summarizes functional and clinical consequences associated with naturally occurring variations in the PAF-AH gene. Many studies have attempted to demonstrate that genetic diversity of PAF-AH affects features related to vascular and other diseases. It is clear that mutations that compromise the normal function of the enzyme (*i.e.*, V279F) increase the incidence and/or severity of several conditions, including vascular disease, in defined populations. Superficially, this relationship may appear contrary to studies showing that *elevated* PAF-AH expression correlates with vascular disease incidence and/or severity in Western populations. However, these two sets of observations can be reconciled from a physiological perspective if increased PAF-AH expression is viewed as a compensatory, not causal, mechanism to limit damage caused by pro-inflammatory phospholipids generated in settings of high oxidant stress. PAF-AH polymorphisms that have more subtle effects in standard laboratory assays have also been reported to modulate severity or incidence of a number of syndromes and diseases. It is possible that evaluating additional enzyme properties, location, and/or functions may help elucidate whether, and how, genetic variations within the coding region of PAF-AH impact its *in vivo* function. The effect of lipoprotein location on the function of PAF-AH remains to be completely elucidated, but the fact that altered distribution has been observed in a variety of human syndromes suggests potentially important effects. This is supported by biochemical studies indicating that the environment provided by LDL allows for higher efficiency of substrate hydrolysis compared to that of HDL. Studies aimed at characterizing whether PAF-AH polymorphisms have a physiological impact should evaluate haplotypes in individual subjects rather than the prevalence of each variant in the sample set studied. This approach could have potential clinical utility as it may help identify patients most likely to benefit from therapies that target this pathway. A number of the studies cited here have used this strategy and it is likely that the approach will become standard practice. SNPs located in the promoter and other regulatory regions of PAF-AH, while not the focus of this review, may have important functional consequences as well. These variants have received limited attention and it would be interesting to evaluate whether they modulate expression levels, and/or the incidence or severity of inflammatory and vascular diseases. To establish meaningful relationships between genotype and function, and to identify the clinical impact of PAF-AH SNPs, it will be necessary to conduct systematic analyses that take into consideration individual genetic complexities, environmental influences, and the unique properties of this interesting enzyme.
